# Anti-tumor effect of polysaccharide from *Pleurotus ostreatus* on H22 mouse Hepatoma ascites in-vivo and hepatocellular carcinoma in-vitro model

**DOI:** 10.1186/s13568-021-01314-5

**Published:** 2021-12-02

**Authors:** Kavish Hasnain Khinsar, Sattar Abdul, Akbar Hussain, Riaz Ud Din, Liu Lei, Jing Cao, Majid Abbasi, Ata Ur Rehman, Nabeel Farooqui, Xin Yi, Huang Min, Liang Wang, Zhong Mintao

**Affiliations:** 1 Department of Microbiology, College of Basic Medical Sciences, Dalian Medical University, Dalian, 116044 China; 2 Department of Biochemistry, College of Basic Medical Sciences, Dalian Medical University, Dalian, Liaoning, 116044 China; 3 Department of Biotechnology, College of Basic Medical Sciences, Dalian Medical University, Dalian, 116044 China; 4 Stem Cell Clinical Research Centre, National Joint Engineering Laboratory, Regenerative Medicine Centre, The First Affiliated Hospital of Dalian Medical University, Dalian, Liaoning, 11601 China; 5 Department of Biochemistry, Ghulam Muhammad Mahar Medical College, SMBB Medical University Larkana, Larkana, Pakistan

**Keywords:** *Pleurotus ostreatus*, Hepatocarcinoma, Malignant ascites, Immunohistochemistry, Polysaccharides

## Abstract

Hepatocellular carcinoma is one of the leading causes of cancer-associated death across the globe. Malignant ascites are the major clinical attributes in cancer patients. Despite the advancements in HCC treatments such as chemotherapy, radiotherapy, surgery, and hormonal therapy, researchers are pursuing novel natural edible compounds for the treatment of cancer to eliminate dreadful side effects. *Pleurotus ostreatus* is one of the most edible cuisines in Asia as well as all over the world. It has been a source of nutritious diet since it was classified as an edible mushroom with no or negligible side effects. The present study focused on the natural anti-cancerous and anti-ascites capabilities of polysaccharides extracted from *Pleurotus ostreatus* in-vivo as well as in-vitro. Administration of polysaccharide *Pleurotus ostreatus* showed a significant decrease in tumor cell metastasis while the increase in the survival period among mice models of H22 malignant ascites. Downregulation of regenerative genes Foxp3 and Stat3 and secretion of immunological factors such as IL-2, TNF α, and INF γ were observed after treating with the partially pure extracted polysaccharide. Twining with the hypothesis of tumor suppression in-vivo model polysaccharide showed a decrease in invasion and migration abilities and henceforth responsible for the gene regulation such Cytochrome C which supposedly induced the chain of gene regulation process resulting in apoptosis in HCC cell lines observed in-vitro experiments. Collective research findings manifested that polysaccharide extracted from *Pleurotus ostreatus* bears anti-proliferative activity and thus influence tumor suppression in-vivo and in-vitro against hepatocellular carcinoma and can be used for therapeutic purposes as a potential anti-cancerous source in the future.

## Keypoints

- *Pleurotus ostreatus* increased survival time period of H22 Malignant Ascites model
- *Pleurotus ostreatus* enhancing the immune response by activating IL2, INFγ, TNFα
- *Pleurotus ostreatus* induced apoptosis in HepG2 and HCCLM3

## Introduction

In recent times, natural products are playing a vital role in strengthening the immune system as well as anti-cancerous agents (Pang et al. 2017). Mushrooms, the basic and rich source of food, contains lower calories and cholesterol and a trace amount of sodium depending upon the type of species in the edible mushroom family (Friedman 2016). *Pleurotus ostreatus* mushroom is one of the most edible and commercially available mushrooms all over the world. *Pleurotus ostreatus* contains one of the most important components involves in the anti-cancer activity of mushrooms i.e., its carbohydrate fraction origin from β-glucans. (Adebayo and Oloke 2017) Polysaccharide fraction of *Pleurotus ostreatus* showed increased secretion of cytokines in an animal model (El Enshasy et al. 2012). β-glucans reported having anti-cancer activity against liver cancer (Pinheiro et al. 2003). β-glucans from *Pleurotus ostreatus* are also used against colon cancer (Lavi et al. 2006).

Polysaccharide is one of the major components of mushrooms proved to have a direct or indirect effect on tumor cell growth. Polysaccharide MP-1 from soybean origin showed direct inhibition of tumor growth (Li et al. 2017). Extracted Polysaccharide from *S. fusiform* was found to have a direct effect against hepatocellular carcinoma (Fan et al. 2017). Inducing apoptosis cell death in HepG2 cells was observed when treated with soybean residue fermented with *Morchella esculenta* (Li et al. 2017). While signaling pathway was affected by the *Astragalus* compound which manifests an anti-tumor effect against Hepatoma. (Wu et al. 2018) Promoting secretions of cytokines *Ganoderma lucidum* showed an anti-tumor effect against H22 transplanted mice. (Chen et al. 2004).

This study aims to examine the anticancerous activity of edible mushrooms and the extraction of crude polysaccharides from *Pleurotus ostreatus* in proposing the mechanism and its function against hepatocellular carcinoma in-vivo as well as in-vitro.

## Materials and methods

### Extraction of polysaccharide from *Pleurotus ostreatus*

*Pleurotus ostreatus* was bought from a local market in Dalian, Liaoning, China. Mushroom (100 g) was dried and ground, then passed through a 40 mesh sieve (0.42 mm) and then dissolved in water with a 1:1 g/L powder-liquid ratio and boiled for 2 h. This mixture was boiled three times with cooling of 6 h at room temperature. The supernatant was then filtered through Whatman grade 1(11 µm) filter paper following by concentrating the liquid by using a rotary evaporator at 60 °C till 95% of water was evaporated. Later, 10% trichloroacetic acid was added in equal volume to precipitate out the protein from the concentrated mixture and incubate it overnight (Koontz 2014). Protein will be settled down in pellet and supernatant was subjected to ethanol precipitation for acquiring polysaccharide. The mixture was incubated at 4 °C for 24–36 h and then centrifuged for 15 min at 3500 rpm (Xu et al. 2014). The supernatant was decanted and the pellet was obtained containing crude polysaccharide. Absolute ethanol and 75% ethanol were used for washing the pellet respectively and centrifuged for 5 min at 8000 rpm. Air dries the pellet and dissolves the crude polysaccharide in de-ionized distilled water. Dialysis with de-ionized distilled water was performed to eliminate impurities. The phenol–Sulphuric acid method was used to quantitate the total carbohydrate content present in a crude mixture (DuBois et al. 1956).

### HCC cell lines and culturing condition

Hepatocarcinoma cell lines HepG2 and HCCLM3 were used against non-malignant LO2 cell lines as control. All cell lines were purchased from Shanghai cell bank, Chinese Academy of Sciences. HCCLM3 and LO2 cell lines were revived and sub-cultured in Dulbecco’s modified Eagle’s medium (DMEM; Gibco, USA) containing 10% fetal bovine serum (ExCell Bio, China). HepG2 was revived and sub-cultured in RPMI-1640 medium (Gibco, USA) with 15% fetal bovine serum (ExCell Bio, China). All the cell lines were grown in the presence of a combination of Penicillin (100 U/ml) and Streptomycin (100 µg/ml) at 37 °C in a humidified incubator with 5% CO_2_. Cells were grown exponentially and passaged when confluency reached up to 70%.

### Cytotoxicity and cell viability assay for HCC cell lines

Cytotoxicity of polysaccharide on HCC cell lines against LO2, cell viability assay was performed. Cell lines were cultured in 96 wells plate separately in the presence of three different concentrations of polysaccharide i.e., 300, 400, and 500 µg/ml, and control was treated with normal saline followed by incubation of plates for 24 and 48 h independently.

After the incubation period, old culture media was removed and 10ul CCK8 solution was added in each well-containing 90 μl of fresh culture media. Plates were incubated for 3–4 h and absorbance was determined on an Enzyme-linked immunosorbent assay ELISA plate reader at 450 nm. Cell viability calculated by the following equation;

$${\text{Cell Viability }}\left( \% \right) = {\text{A }}\left( {\text{Experimental group}} \right)/{\text{B }}\left( {\text{Control group}} \right) \times {1}00$$

### RNA extraction and quantitative real-time polymerase chain reaction (qRT-PCR)

To determine the expression of genes involved in apoptosis and cell death quantitative real-time polymerase chain reaction qRT-PCR was performed. Following treatment with three concentrations of polysaccharide (300, 400, and 500 µg/ml) total RNA was extracted from hepatic cell lines by using Trizol™ (Invitrogen, USA) reagent according to manufactures instruction. cDNA was synthesized using RNA by using a Transgene kit. Transcripts were quantified by StepOne™ 7300 plus applied biosystems (Life, USA) machine by using SYBER green master mix kit (Transgene). The comparative cycle threshold 2^−ΔΔCT^ was used to calculate the relative expression. GAPDH was used as endogenous control and experiments were repeated three times independently.

The sequences of primers used are enlisted in Table 1.

**Table 1:** Primer sequences used in the qPCR experiment

GENE	FORWARD 5ʹ → 3ʹ	REVERSE 5ʹ → 3ʹ
Apaf-1	AGTGGCAAGGACACAGATGG	GGCTTCCGCAGCTAACACA
Bcl-2	GGTGGGGTCATGTGTGTGG	CGGTTCAGGTACTCAGTCATCC
Caspase3	AGAGGGGATCGTTGTAGAAGTC	ACAGTCCAGTTCTGTACCACG
Caspase9	CTCAGACCAGAGATTCGCAAAC	GCATTTCCCCTCAAACTCTCAA
GAPDH	AATCCCATCACCATCTTCCA	TGGACTCCACGACGTACTCA
Cytochrome C	GCCTCAGAATAACCATTGTCC	TCAAATACCCCAAACTCCAAC

### Colony formation assay

1 × 10^3^/per ml Polysaccharide-treated HCCLM3 and HepG2 were seeded in six-well plates containing respective cultured medium. Cells were incubated for 8–10 days at 37 °C in a humidified atmosphere with 5% CO_2_. Fresh medium was replaced every 3–4 days (if necessary). Once the colonies were visible with the naked eye, fixed with 4% formaldehyde for 10–15 min. And then washed gently with ice-cold PBS 2 times. Crystal violet (0.5%) was then added for 30 min at RT (room temperature) to stain colonies. Excess stain was removed by gentle washing with PBS 3–4 times. Photographs were taken after colony observation.

### Migration and invasion assay

Migration and Invasion assay of HCCLM3 and HepG2 were carried out by culturing HCCLM3 in DMEM and HepG2 in RPMI-1640 and treating with the concentrations of polysaccharide as in the above experiments. For invasion assay, 100 µl matrigel (prepared in 1:50 dilution in serum-free culture media) was added in each chamber and incubated in an incubator at 37 °C with 5% CO_2_. For migration assay, the same protocol was followed except for the addition of matrigel. The next day, 600 µl serum-containing medium was added to the lower chamber and the upper chamber contains cells (2.5 × 10^5^/ml cells) in 200 µl serum-free culture medium placed in 24- wells plate. Plates were incubated in the humidified container with 5% CO_2_ at 37 °C for 12–16 h. After removing the culture media from chambers and washing with PBS, cells were fixed with 4% formaldehyde for 10–15 min. After that formaldehyde was removed and cells were washed with PBS and then 0.5% crystal violet was covered for 15 min. The stain was removed and the chamber was washed with PBS 2–3 times. The migrated and invaded HCC cells were observed and their images were captured under the microscope (Olympus, Japan) with 20 magnification.

### Wound healing assay

2.0 × 10^5^ per ml HCCLM3 and HepG2 treated cells with polysaccharide were plated in six-well plates and placed in humidified containers with 5% CO_2_ at 37 °C for 12–16 h. Then scratch was made with the sharp sterile object on the cell monolayer. Floated cells were discarded and a fresh medium was added and then the plate was incubated for 48–72 h in a 5% CO_2_ incubator. A relative distance gap was noted at an interval of 0, 24, 48, and 72 h using image J software.

### Western blot

Polysaccharide-treated HCC cells and control cells were collected and washed twice with ice-cold PBS buffer. Cells were lysed with RIPA (Transgene, Biotech, Beijing, China) buffer, containing phosphate and protease inhibitors, for 30 min on ice. The lysate was centrifuged at 12000 rpm at 4 °C for 15 min. The supernatant was collected in a fresh tube and protein concentration was measured by the BCA protein quantitation assay kit (KeyGEN BioTECH). Proteins (20–40 µg) were resolved by electrophoresis and transferred onto the PVDF (Polyvinylidene fluoride) membrane (Millipore, Billerica, MA, USA). Non-specific binding was avoided by incubating membrane in a blocking solution containing 5% skimmed milk in TBST (20 mM Tris–HCl pH 7.5, 150 mM NaCl, 0.1% Tween 20) for 2 h in room temperature. Blots were incubated at 4 °C overnight with primary antibodies Bcl-2 (1:1000, Affinity Biosciences, USA), Cytochrome C (1:500, Affinity Biosciences, USA), APAF1 (1:1000, Affinity Biosciences, USA) Caspase 3 (1:1000, Affinity Biosciences, USA), Caspase 9 (1:1000, Affinity Biosciences, USA) Stat3 (1:1000, Affinity Biosciences, USA), Foxp3 (1:1000, Affinity Biosciences, USA) and GAPDH (1:4000, Proteintech, USA). The next day, blots were washed with TBST three times and the membrane was incubated with secondary antibody HRP conjugated goat anti-rabbit IgG or goat anti-mouse IgG (Proteintech, USA) for one hour at room temperature. ECL (Enhanced Chemiluminescence) kit was used for visualization in ChemiDcoTM XRS + Imager-Bio-Rad and images were captured and saved.

### Immuno-florescence assay

2 × 10^5^ HCCLM3 cells per ml were cultured and treated with polysaccharide after 48 h of incubation and were washed twice with PBS. Cells were fixed with 4% formaldehyde for 20 min at RT. Then washed with ice-cold PBS to remove formaldehyde. Triton X-100 (0.1%) was used to cause cell penetration at RT for 10 min. Cells were then washed with PBS three times for 5 min per wash. Blocking was done by 300 µl of 0.3% BSA at RT for 60 min. Cytochrome C antibody was added with the dilution of 1:200 and incubated at 4 °C in dark. The antibody was removed by PBS washing 3 times. After that, 200 µl of 10 µg/ml DAPI (4ʹ, 6-diamidino-2-phenylindole) was added at RT for 10 min in dark. DAPI was removed by PBS washing three times for 5 min. Images were captured using a fluorescence microscope (Olympus, Japan) with a magnification of 20X.

### Anti-tumor activity of *Pleurotus ostreatus* polysaccharide in-vivo

#### Establishing H22 bearing mice model

BALB/c mice were obtained from specific pathogen-free (SPF) houses (Dalian Medical University, Dalian, China). H22 cells in the logarithmic growth phase were adjusted as 1 × 10^6^ cells /ml in trypane blue stain with 95% viability. Cells were washed with 0.85% saline 2–3 times and then tumor cells in 0.2 ml of saline suspension were injected into the left peritoneal cavity of BALB/c mice. 40 mice were randomly divided into 5 groups. Groups 1–5 were organized as: Control group, 75 mg/kg, 150 mg/kg, 300 mg/kg and CTX (Cyclophosphamide group, 20 mg/kg) in 0.85% saline.

#### The survival rate of mice bearing H22 mouse hepatoma ascites model

To observe the survival period among all the groups of mice, all groups were injected with their designated doses of polysaccharide and CTX. Polysaccharide and drug administration were performed by intraperitoneal injection on the left side of each mouse. From the day of injecting the tumor cells till all the mice died, the data was recorded for bodyweight, the abdominal circumference of each mouse, along with strict observation on food and water consumption.

#### Sacrifice and tissue specimen collection from H22 mouse hepatoma ascites model

Another set of 40 mice were purchased from SPF (Dalian Medical University, Dalian, China). Following intraperitoneal injection of H22 cells, all the groups were administered with drugs with their respective concentration and finally were sacrificed after 15 days. Blood and liver tissues were collected. A blood sample was kept at 4 °C for 30 min and serum was separated by centrifugation at 1400 rpm for 10 min at 4 °C and also stored at − 80 °C for further experimentation. Tissue samples were washed in sterile chilled PBS and preserved in 4% formaldehyde at 4 °C for further use.

#### Cytokines level in serum of H22 mouse hepatoma ascites model

Competitive Enzyme-linked immunosorbent assay (ELISA) was carried out to evaluate the quantitative analysis of Interleukin-2 (IL-2), Tumor Necrosis Factor α (TNF- α), and Interferon-gamma (INF-γ). Blood samples were placed at room temperature for 10–20 min then centrifuged for 20 min (2000–3000 rpm) at 4 °C and supernatant was collected in a sterile tube. ELISA kit reagents (Shanghai, Langdun Biosciences. Ltd) were pre-incubated for 30 min at room temperature before the experiment. All the chemicals were reconstituted according to the manufacturer’s protocol. A serial dilution procedure was used for making standard references. Biotinylated Antigen and Avidin-HRP were also diluted according to the manufacturer’s guidelines. 50 µl diluted reference standard was added in each well and 50 µl of reference was added in zero well (Zero well will be used as the last point of standard curve). 50 µl sample was inoculated in the sample well. Then 50 µl of working solution of Biotinylated antigen was added in all the references and serum sample wells. The plate was covered with a plate sealer and incubated at 37 °C for 30 min. After careful removal of plate sealer, the liquid was thrown away and feeding all wells with a working concentration of wash buffer and keeping plate for 30 s and the liquid was abandoned. The procedure was repeated 5 times. 50 µl Avidin-HRP was added in reference as well as in sample wells and swayed tenderly, and the plate was covered with a plate sealer and then incubated in a 37 °C incubator for 30 min. For the second washing removing the plate sealer and the liquid was thrown away, the plate was dried, wash buffer was added, the plate was kept for 30 s and the liquid was abandoned. The procedure was repeated 5 times. 50 µl colored substrate A was added to each well and then 50 µl colored substrate B was added, swayed, and mixed tenderly avoiding light for 10 min. 50 µl stop solution was added to each well to stop the reaction. Blank zero was employed for setting and measuring absorbance for each well under 450 nm wavelength (measure within 10 min after adding stop solution). The regression equation of the standard curve was calculated according to the concentration and OD value of samples.

### Immunohistochemistry for the expression of Foxp3 and Stat3 in liver tissues

Freshly dissected tissues (approximately 3 mm thickness) were fixed with 10% formalin for 24–48 h at room temperature. After fixation, the tissues were rinsed under running water for 1 h. The tissues were dehydrated using 70% alcohol for 45 min then transferred tissues to 80% and 95% alcohol, respectively, each for 45 min, followed by 3 washes with 100% alcohol, replacing the solution every hour. Place the tissues in xylene for 1 h and change the xylene solution after every 30 min. Tissues were embedded in a paraffin block and can be stored at room temperature for years. Paraffin-embedded tissue was sectioned with a thickness of 5–8 µm on a microtome and kept at 40 °C inside a water bath containing distilled water. Floating tissues were transferred on clean glass slides, allowing the slides to dry overnight and kept at room temperature until ready to use.

*Deparafinized the slides in xylene*; repeat the procedure 2 times, 5 min each. *Antigen retrieval* For antigen retrieval 10 mM citric acid buffer (PH 6.0) is used. Heat the solution at medium heat for 8 min avoiding excessive evaporation of the buffer during the process and never dry the tablets. After being allowed to cool, the slides were placed in PBS (PH 7.4) and washed on a decolorizing shaker 3 times for 5 min each. The endogenous peroxidase activity was blocked by incubating the sections in 3% hydrogen peroxide solution and incubated them at RT for 25 min in the dark, placing the slides in PBS (PH7.4), and washing those 5 × 3 min. *Serum blocking*: 3% BSA was added drop-wise in the histo-chemical circle to cover the tissue uniformly, at room temperature for 30 min (Primary antibodies are blocked with rabbit serum and other sources are blocked with BSA). Primary antibody was added drop-wise to the section and place section in a wet box incubated at 4 °C overnight. The next day, a secondary antibody was added after washing with PBS (PH7.4) for 3 × 5 min. After the sections were slightly dried, the secondary tissue (HRP-labeled) was added drop-wise in the circle, and the tissue was incubated at room temperature for 50 min. For DAB (3,3′-Diaminobenzidine), color development slides were placed in PBS (PH 7.4) and washed 3 times for 5 min each. After the sections were slightly dried, freshly prepared DAB (Diaminobenzidine) color developing solution was added drop-wise in the circle. The color development time was controlled under the microscope. The sections were washed with tap water to stop the color development.

### Western blot from the tissue samples

Tissue samples were cut on a glass plate/ slide and kept on dry ice (20-50 mg of the tissue sample is good enough). 20 µl/mg of RIPA buffer was added and homogenized with a hand-held homogenizer inside a glass test tube in the presence of RIPA buffer until the tissue sample is homogenized completely. Homogenized solution was transferred in a clean Eppendorf tube and centrifuged in pre-cool high-speed microfuge at 14,000 rpm at 4 °C for 10 min. The supernatant was removed and kept in a clean Eppendorf tube at − 80 °C for further use in western blot. The next protocol of quantification and running western blot protocol is the same as other protein western blot protocol.

### Statistical analysis

Graph Pad Prism version 6.0 software was used for data analysis. Data was represented and expressed as (Mean ± SD). Student’s t-test and one-way analysis of variance (ANOVA) were used for data analysis, and the statistical p-value was set as *p < 0.05, **p < 0.01, and ***p < 0.001, NS means no statistical significance. All the experiments were carried out in triplicate independently or otherwise mentioned.

## Results

### Effects of polysaccharide on the survival rate of Murine Hepatocarcinoma

**Fig. 1:**
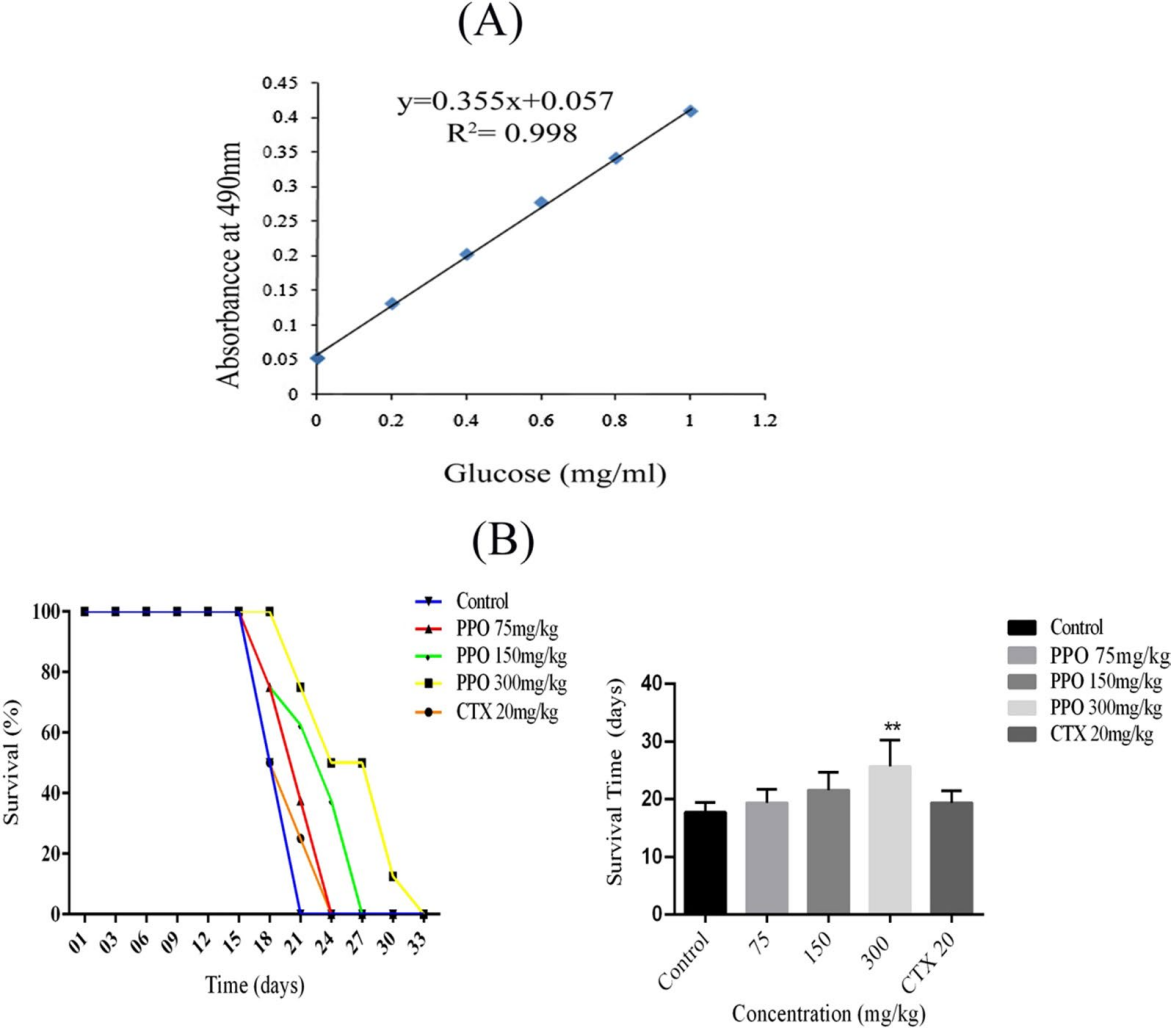
Polysaccharide extracted from *Pleurotus ostreatus* **A** Standard curve used to quantitate total carbohydrate component in extract **B** Murine hepatocarcinoma H22 malignant ascites models were established to observe survival time of mice models after treating with polysaccharide and results shows dose-dependent increase in survival period *p < 0.05, **p < 0.01, ***p < 0.001. (n = 40) *PPO (Polysaccharide from *Pleurotus ostreatus*), *CTX (Cyclophosphamide)

The final concentration of partially purified polysaccharide extracted from *Pleurotus ostreatus* was measured 40 mg/ml by using d-glucose as standard and calculated by using the equation as shown in Fig. 1A. The highest survival time was observed at 33 days with the polysaccharide concentration of 300 mg/kg and the lowest survival period was observed at 21 days in the control group. It was observed that 75 mg/kg concentration of polysaccharide and CTX group have almost the same survival time, whereas 150 mg/kg concentration of experimental group exhibited the best survival against control, 75 mg/kg and CTX concentration group but in comparison, it is lower than 300 mg/kg concentration group as shown in Fig. 1B (*p < 0.05, **p < 0.01, ***p < 0.001).

### Anti-tumor activity of PPO against Hepatocarcinoma H22 Ascites model in BALB/c:

**Fig. 2:**
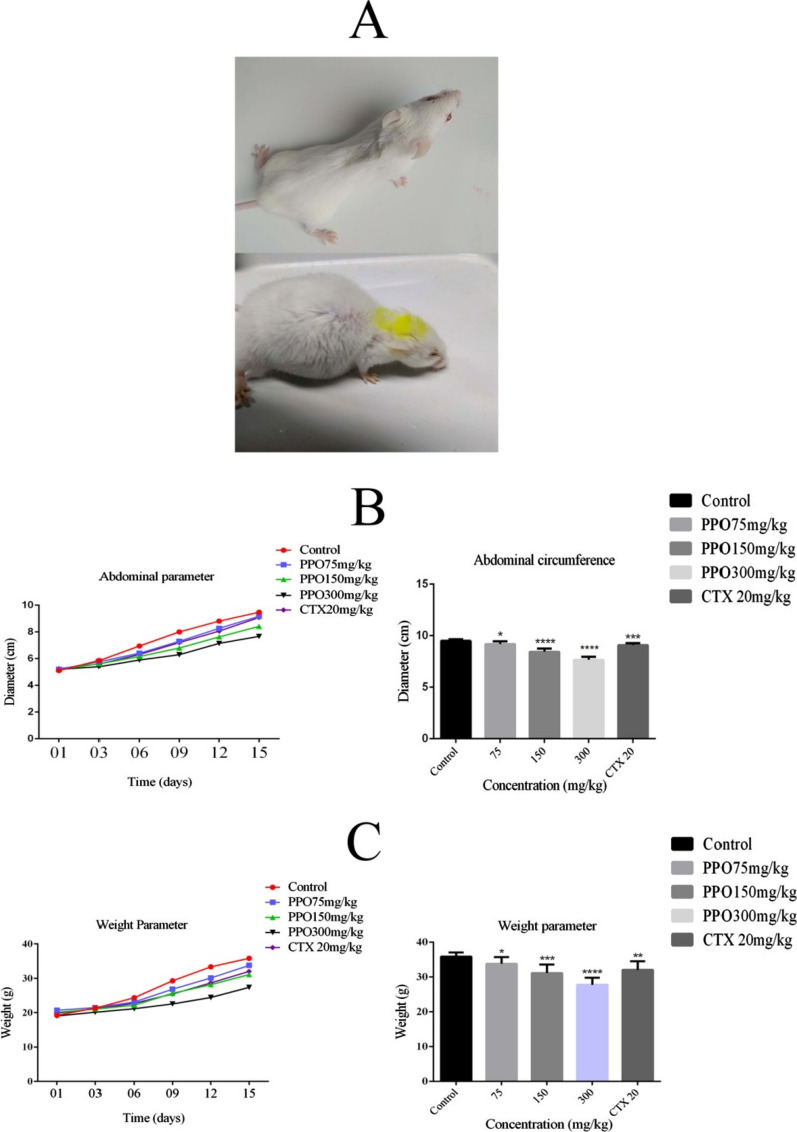
The effects of H22 malignant ascites on mice model after treating with polysaccharide **A** Mice photographed before and after developing the ascites. **B** The abdominal perimeter was observed for 15 days and results suggested that there is a significant difference between Control and polysaccharide treated group. **C** Weight perimeter was also observed for 15 days and shows the significant difference between Control and polysaccharide treated group in a dose-dependent manner. *p < 0.05, **p < 0.01, ***p < 0.001. (n = 40) *PPO (Polysaccharide from *Pleurotus ostreatus*), *CTX (Cyclophosphamide)

Figure 2A shows the significant differences in abdominal diameter in treatment groups versus control group as 19.30% (***p < 0.001) and 10.6% (***p < 0.001) in polysaccharide concentration of 300 mg/kg and 150 mg/kg respectively, while 75 mg/kg and CTX shows almost the same and little differences as 3.2% (*p < 0.05) and 4.21% (***p < 0.001) against control group respectively. Figure 2B demonstrate the significant differences in the weight of mice in the concentration group of 300 mg/kg, 150 mg/kg, 75 mg/kg and CTX that are 22.45% (***p < 0.001), 13.12% (***p < 0.001), 5.86% (*p < 0.05) and 10.58% (**p < 0.01), respectively, against control group.

### Foxp3 and Stat3 expression by IHC and western blot in vivo; and Cytokines expression in mice blood serum

**Fig. 3:**
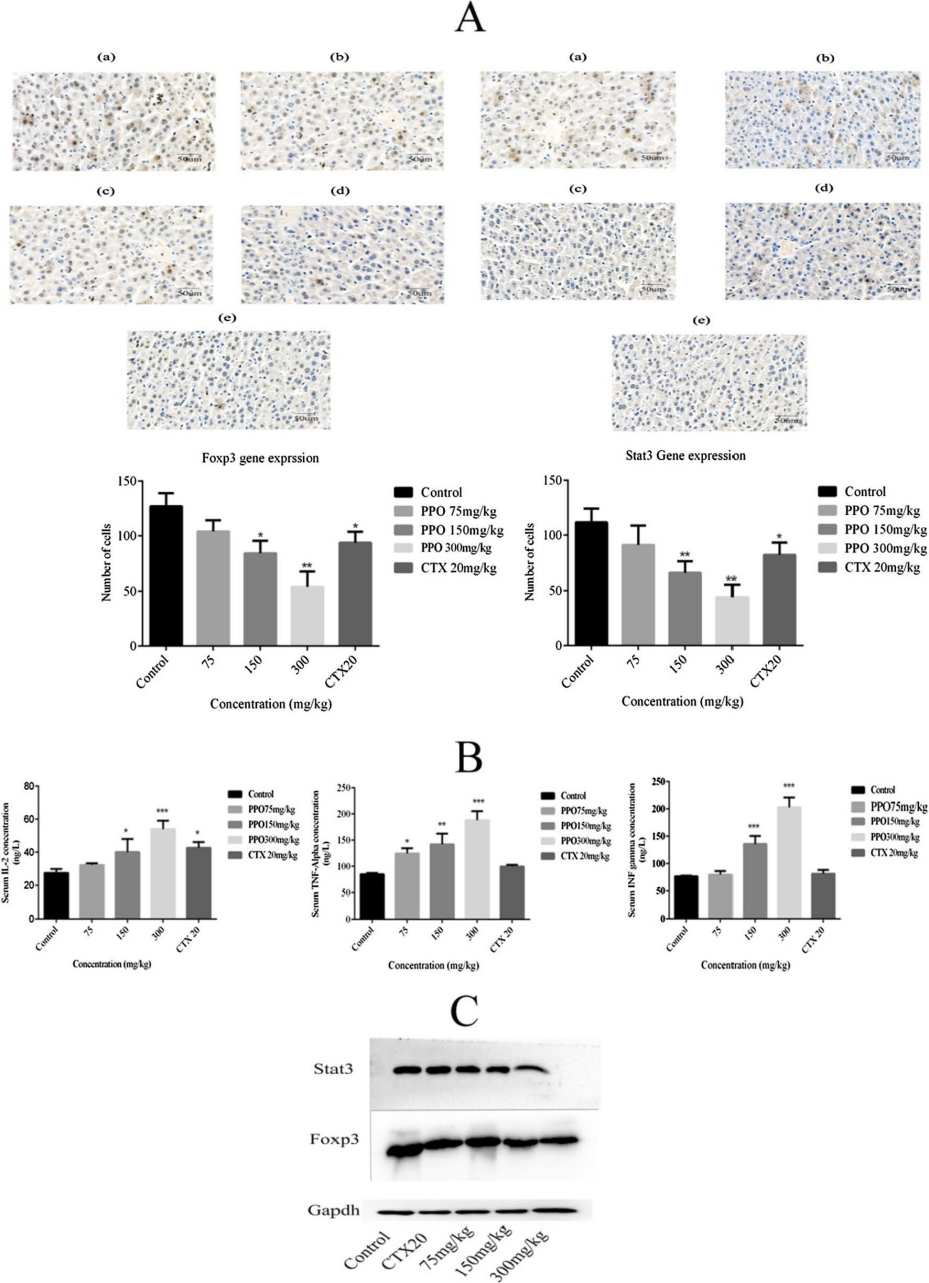
Immunomodulatory effects of polysaccharide on H22 malignant ascites mice model **A** Immunohistochemistry was performed of liver sample tissues to observed Foxp3 and Stat3 expression in mice model and observed a significant decrease in gene expression in a dose-dependent manner. Images were captured at the 40X lens. **B** Cytokines level was checked by performing Elisa such as IL-2, TNFα, and INFγ was observed to have a significant increase in serum in a dose-dependent manner. **C** Western blot analysis was performed to observe protein expression of Foxp3 and Stat3 and results show a significant dose-dependent decrease in expression. *p < 0.05, **p < 0.01, ***p ≤ 0.001. *PPO (Polysaccharide from *Pleurotus ostreatus*), *CTX (Cyclophosphamide)

Immunohistochemistry results showed the decrease in the number of cells and decrease in gene expression of Foxp3 in concentration group of 300, 150, 75 mg/kg and CTX that are 57.48% (**p < 0.01), 33.85% (*p < 0.05), 18.11% (ns) and 26.29% (*p < 0.05), respectively, comparing with control group. Whereas decreased gene expression of Stat3 was observed in treatment group of 300, 150, 75 mg/kg and CTX as 60.57% (**p < 0.01), 41.21% (**p < 0.01), 18.45% (ns) and 26.52% (*p < 0.05), respectively, against control group as shown in Fig. 3A.

Expressions of IL-2 was found to be increased by 15.2% (ns), 31.72% (*p < 0.05), 50.76% (***p < 0.001) and 35.81% (*p < 0.05) in group of 75, 150, 300 mg/kg and CTX, respectively. INF γ was increased by 5% (ns), 43.98% (ns), 62.29% (***p < 0.001) and 5.91% (ns) in treatment group of 75,150,300 mg/kg and CTX, respectively. TNF α were increased by 31.94% (*p < 0.05), 40.28% (**p < 0.01), 54.90% (***p < 0.001) and 14.88% in 75,150,300 mg/kg and CTX group, respectively, shown in Fig. 3B. Western blot analysis also confirmed the decresed in gene expression of Foxp3 and Stat3 in a dose-dependent manner (Fig. 3C).

### Anti-tumor activity of Polysaccharide extracted from *Pleurotus ostreatus* in-vitro

#### Effects of polysaccharide on cell viability and colony formation against HCC

**Fig. 4:**
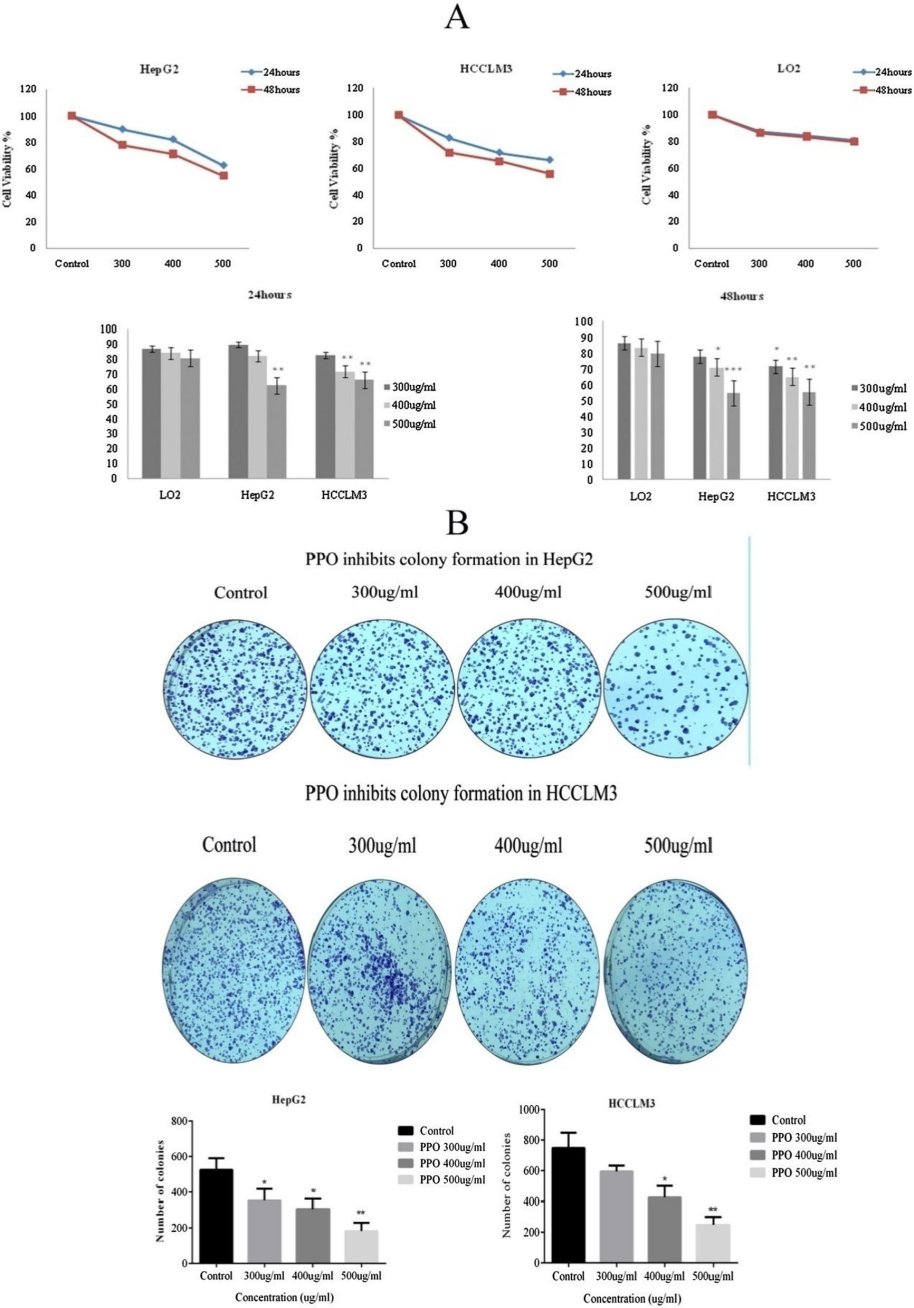
Polysaccharide effects were observed in-vitro using HCC cell lines **A** Cytotoxicity cell viability assay shows the effects of polysaccharide on LO-2, HepG2, and HCCLM3 cell lines. In comparison to normal cell lines, cancer cell lines showed a decrease in cell viability at 24, 48 h with three different concentrations 300, 400, and 500 µg/ml. **B** Polysaccharide inhibits colony formation abilities of cancer cells after treating for 48 h and showed a significant decrease in colony formation abilities of HepG2 and HCCLM3 cell lines in a dose-dependent manner. *p < 0.05, **p < 0.01, ***p ≤ 0.001. *PPO (Polysaccharide from *Pleurotus ostreatus*), *CTX (Cyclophosphamide)

In comparison with LO2, cell viability of HepG2 and HCCLM3, having 300 µg/ml concentration of polysaccharide after 24 h, was decreased slightly whereas after 48 h cell viability of HepG2 and HCCLM3 was decreased by 8.37% and 14.62% (*p < 0.05), respectively. With a concentration of 400 µg/ml, HepG2 and HCCLM3 showed a 1.96% and 12.36% (**p < 0.01) decrease in viability at 24 h respectively. While at 48 h, 12.42% (*p < 0.05) in HepG2 and 18.62% (**p < 0.01) in HCCLM3 showed suppression in growth of cells. Similarly, at 500 µg/ml concentration of extracted polysaccharide, 18.36% (**p < 0.01) in HepG2 and 14.56% (**p < 0.01) in HCCLM3 decrease in viability was recorded at 24 h whereas 24.98% (***p < 0.001) in HepG2 and 24.13% (**p < 0.01) in HCCLM3 showed decrease in viability after 48 h (Fig. 4A).

In comparison with the control group, 300 µg/ml concentration showed a 20.65% and 32.8% (*p < 0.05) decrease in colony formation in HCCLM3 and HepG2 cells respectively. 400 µg/ml concentration of polysaccharide showed a decrease of about 43.26% (*p < 0.05) in HCCLM3 and 41.65% (*p < 0.05) in HepG2 as compared to the control group. At 500 µg/ml concentration of polysaccharide we observed decrease in colony formation of HCCLM3 by 66.75% (**p < 0.01) and 65.08% (**p < 0.01) of HepG2, as compare to control group (Fig. 4B).

#### Anti-metastatic, anti-migratory, and anti-invasive activity of polysaccharide against HCC

**Fig. 5:**
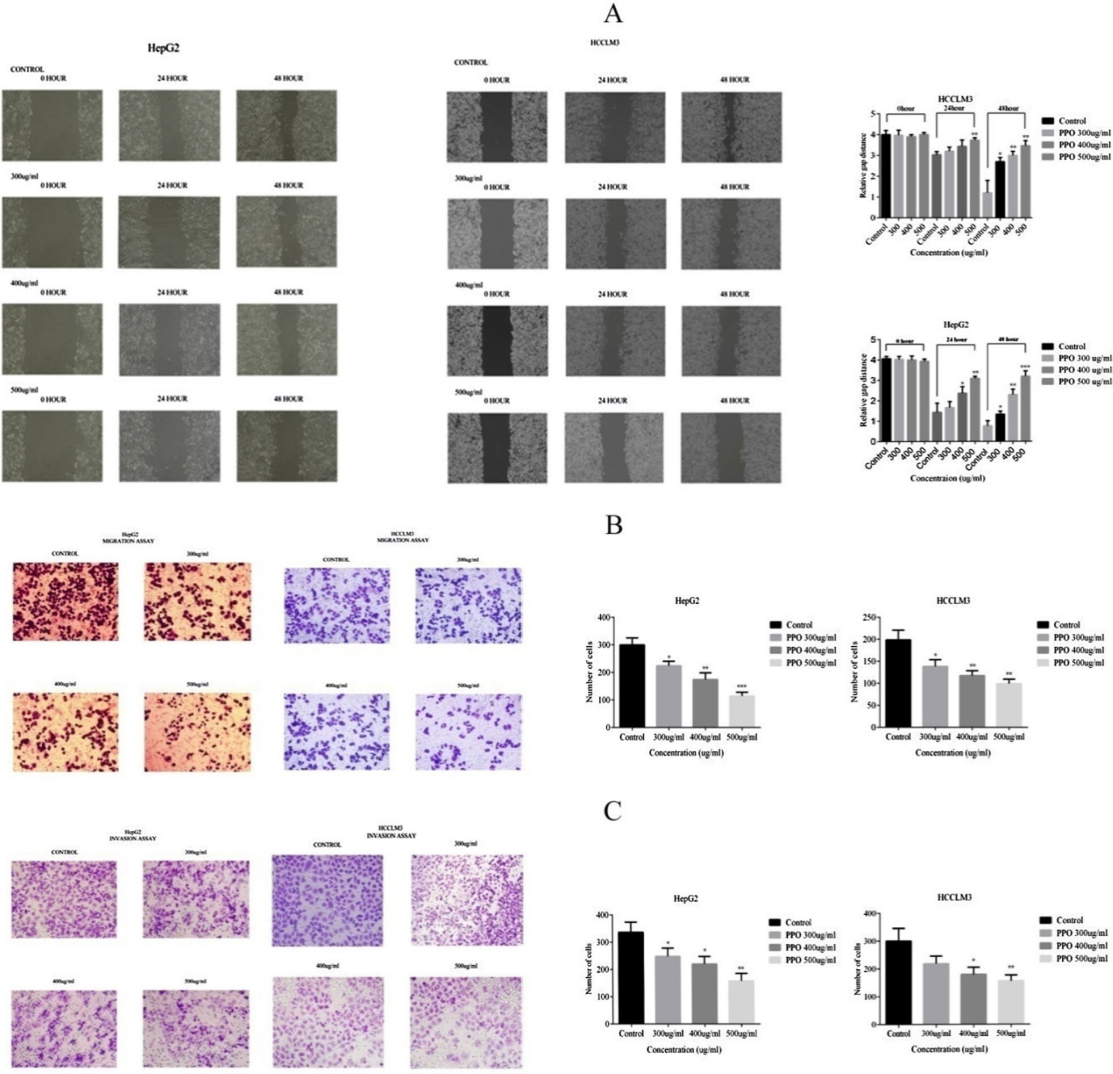
Polysaccharide affects anti-metastatic abilities of cancer cells **A** Wound closure assay was performed and observe the significant difference between Control and polysaccharide treated cells after comparing relative gap distance in HepG2 and HCCLM3. **B** To observe the anti-migratory abilities of polysaccharide in HepG2 and HCCLM3, transwell chambers assay was performed and the difference was observed in decrease number of migrated cells in a dose-dependent manner **C** Transwell chamber was used for invasion assay and the results suggest that there is a significant difference as compared to control group and observed a decrease in the invaded number of cells in a dose-dependent manner. *p < 0.05, **p < 0.01, ***p ≤ 0.001. *PPO (Polysaccharide from *Pleurotus ostreatus*), *CTX (Cyclophosphamide)

In wound closure, significantly decreased results of cell mobility were observed after 48 h at 300 µg/ml in HepG2 and HCCLM3 by 41.53% (*p < 0.05) and 55% (*p < 0.05), respectively. With 400 µg/ml concentration of polysaccharide we found a decrease in cell mobility by 66.95% (**p < 0.01) in HepG2 and 60% (**p < 0.01) in HCCLM3 comparing with control. In the highest dose of polysaccharide at 500 µg/ml, 76.25% (***p < 0.001) and 65% (**p < 0.01) decrease was observed in cells migratory abilities of HepG2 and HCCLM3 respectively when compared with control as shown in Fig. 5A.

Transwell chamber results demonstrated that migration property of HepG2 was decreased by 25.33% (*p < 0.05), 42% (**p < 0.01) and 62% (***p < 0.001) at 300, 400 and 500 µg/ml concentration of polysaccharide respectively. HCCLM3 showed 30.38% (*p < 0.05), 40.93% (**p < 0.01) and 50.01% (**p < 0.01) decrease in cell migration with 300, 400 and 500 µg/ml concentration, respectively, compared with their control as shown in Fig. 5B.

Similarly, invasive properties of HCCLM3 was decreased by 27.2%, 40.33% (*p < 0.05) and 48.13% (**p < 0.01) treating with 300, 400 and 500 µg/ml concentration of polysaccharide respectively, compared with control. And HepG2 showed 26.23%, 34.64% (*p < 0.05) and 53.06% (**p < 0.01) decreased invasive property when treating with 300, 400, and 500 µg/ml polysaccharide concentrations respectively, compared with control, as shown in Fig. 5C.

#### Relative mRNA expression and Immunofluorescence for Cytochrome C expression in HCC

**Fig. 6:**
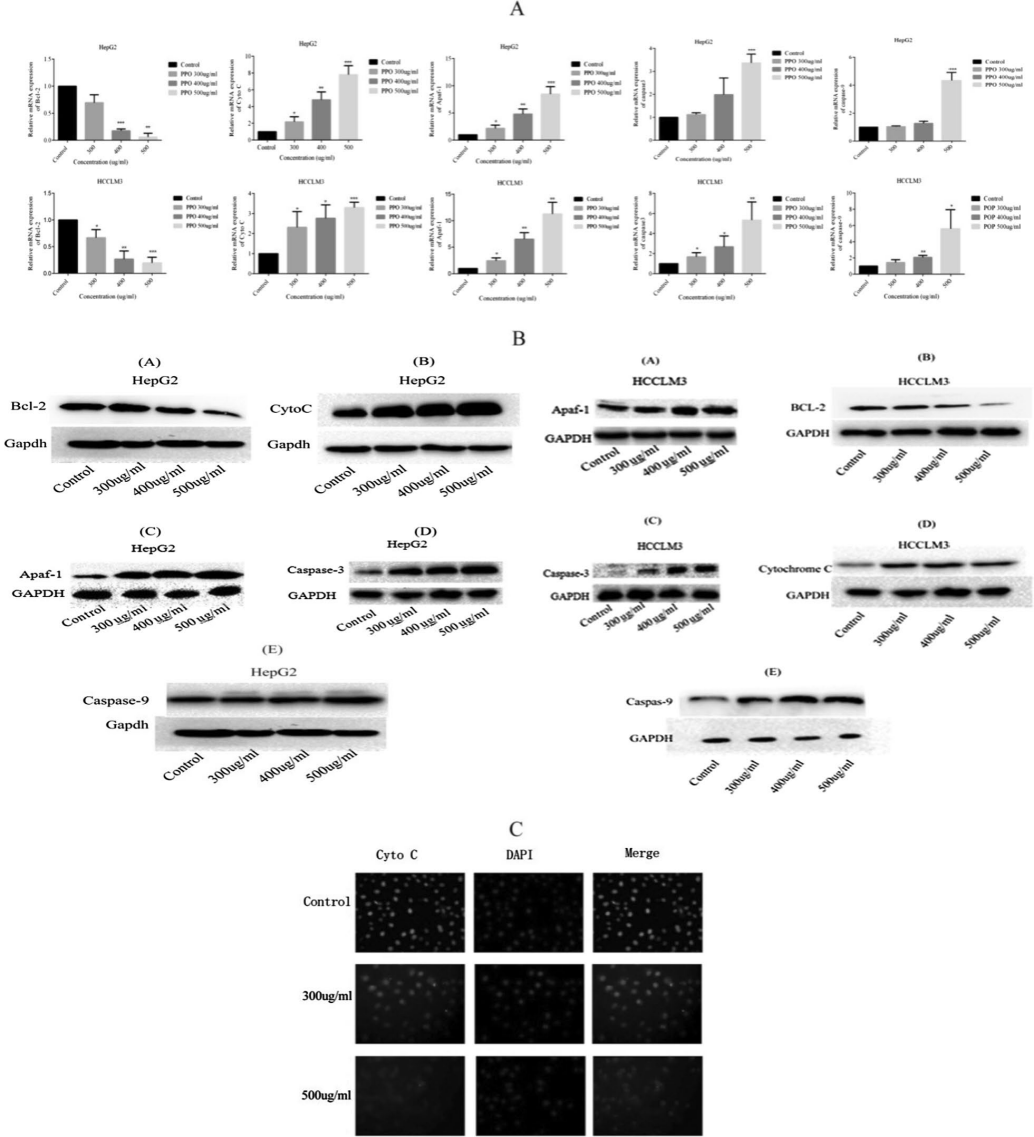
Gene expression of genes involved in cell death was quantified **A** qPCR was performed to observe gene expression of five different genes in both HepG2 and HCCLM3. **B** Western blot assay was performed to observe the expression of five genes including BCL-2, Cytochrome c, Apaf-1, Caspase-9, and Caspase-3. **C** Immunofluorescence assay was performed to observe gene expression of Cytochrome c as it was supposed to be the initiator of apoptosis. *p < 0.05, **p < 0.01, ***p ≤ 0.001. *PPO (Polysaccharide from *Pleurotus ostreatus*), *CTX (Cyclophosphamide)

In HepG2, Bcl-2 down regulated significantly with concentration of 400 and 500 µg/ml that is 83% (***p < 0.001) and 94% (**p < 0.01) respectively, compared with control. In HCCLM3 decrease in expression was observed by 74% (**p < 0.01) and 81% (***p < 0.001) at 400 and 500 µg/ml concentrations, respectively. Cytochrome C expression was increased in HepG2 at 400 and 500 µg/ml concentrations by 79.16% (**p < 0.01) and 87.17% (***p < 0.001) respectively, 62.96% (*p < 0.05) and 69.69% (***p < 0.001) increased expression in HCCLM3 respectively. Apaf-1 was significantly increased in HepG2 by 79.16% (**p < 0.01) and 88.09% (***p < 0.001) at 400 and 500 µg/ml, respectively, where as in HCCLM3 by 84.61% (**p < 0.01) at 400 µg/ml and 91.11% (**p < 0.01) at 500 µg/ml was observed. Significantly increased expression of Caspase3 by 70.23% (***p < 0.001) at only 500 µg/ml was observed in HepG2 cells and increased expression of caspase 3 by 63.76% (*p < 0.05) at 400 µg/ml and 81.13% (**p < 0.01) at 500 µg/ml was observed in HCCLM3. Highest concentration of polysaccharide significantly increased the expression of Caspase9 in both HepG2 and HCCLM3, by 76.90% (***p < 0.001) and 82.14% (*p < 0.05), respectively, as shown in Fig. 6A.Western blot analysis results showed same pattern of protein expression as shown in Fig. 6B.

Expression of Cytochrome C in HCCLM3 by immunofluorescence assay has highlighted the release of Cytochrome C which then supposedly activated the other protein and cause cell death (Fig. 6C).

## Discussion

*Pleurotus ostreatus* is the most popular mushroom across the world and beyond its taste and aroma, it is known as one of the most potent mushrooms having therapeutic credentials. The polysaccharide is one of the major compositional characters of the mushroom fruiting body. Polysaccharides in the past decade have been used as a therapeutic agent and many of them have already been in a process of clinical trials (Cheng et al. 2015; Tian et al. 2016; Yan et al. 2017). Polysaccharide extracted from *Pleurotus ostreatus* was extracted to elucidate its potential as a therapeutic agent. (Liao et al. 2015). The main idea behind these biological experiments was to observe the efficacy of the polysaccharide if used as a potent anti-tumor drug, as there are no extensive studies done on this polysaccharide against hepatocellular carcinoma. In-vivo experiments polysaccharide showed a significant increase in survival time of mice model as shown in Fig. 1B that supports our hypothesis of polysaccharide being used as an adjuvant in chemotherapy or immunotherapy as it can increase the survival time to overcome the metastasis in cancer patients. In -vivo studies show that after the administration of polysaccharide fluid accumulation in the H22 malignant ascites model decreases significantly as shown in Fig. 2 that reflects the anti-ascites attributes of our extracted polysaccharide. Re-occurrence of tumor cells is one of the crucial hurdles in achieving successful cancer therapy (Fang et al. 2018). Foxp3 and Stat3 genes were observed down-regulated in polysaccharide treated groups as compared to their control group that shows no signs of disease re-occurrence results presented in Fig. 3A. Historically, mushrooms were categorized as an immune-boosting diet and play a critical role as an anti-cancerous agent (Prakken et al. 2007; Schrama et al. 2017). Traditionally polysaccharides were assumed to be the immunological modulators and certainly our result which shows increased cytokine levels in Fig. 3B suggests that the polysaccharide can be classified as an immunological modulator.

Furthermore, the in-vitro investigation related to the anti-tumor activity of polysaccharides against hepatocellular carcinoma cell lines (non-cancerous) LO2 was used against HepG2 and HCCLM3 shown in Fig. 4A to observe the toxicity of polysaccharide used (Kang et al. 2016). Anti-tumor activities of polysaccharides were observed against different phenotypic attributes of cancerous cells such as their colony formation, metastatic and migration abilities results shown in Fig. 4B. Colony formation of cancer cells affects as the cells grow rapidly and form clusters which eventually cause cirrhosis (Ohki et al. 1988). Metastasis of cancer cells is also one of the causes of mortalities as they have metastasized and invade other tissues (Hanahan and Weinberg 2000). Observed results in Fig. 5 show HCC cell lines were susceptible to polysaccharides from *Pleurotus ostreatus*. The most foundational feature of metastasis is migration and invasion (Chen et al. 2014). Applied polysaccharides attenuated the metastasis activity of cancer cells in a dose-dependent manner.

**Fig. 7:**
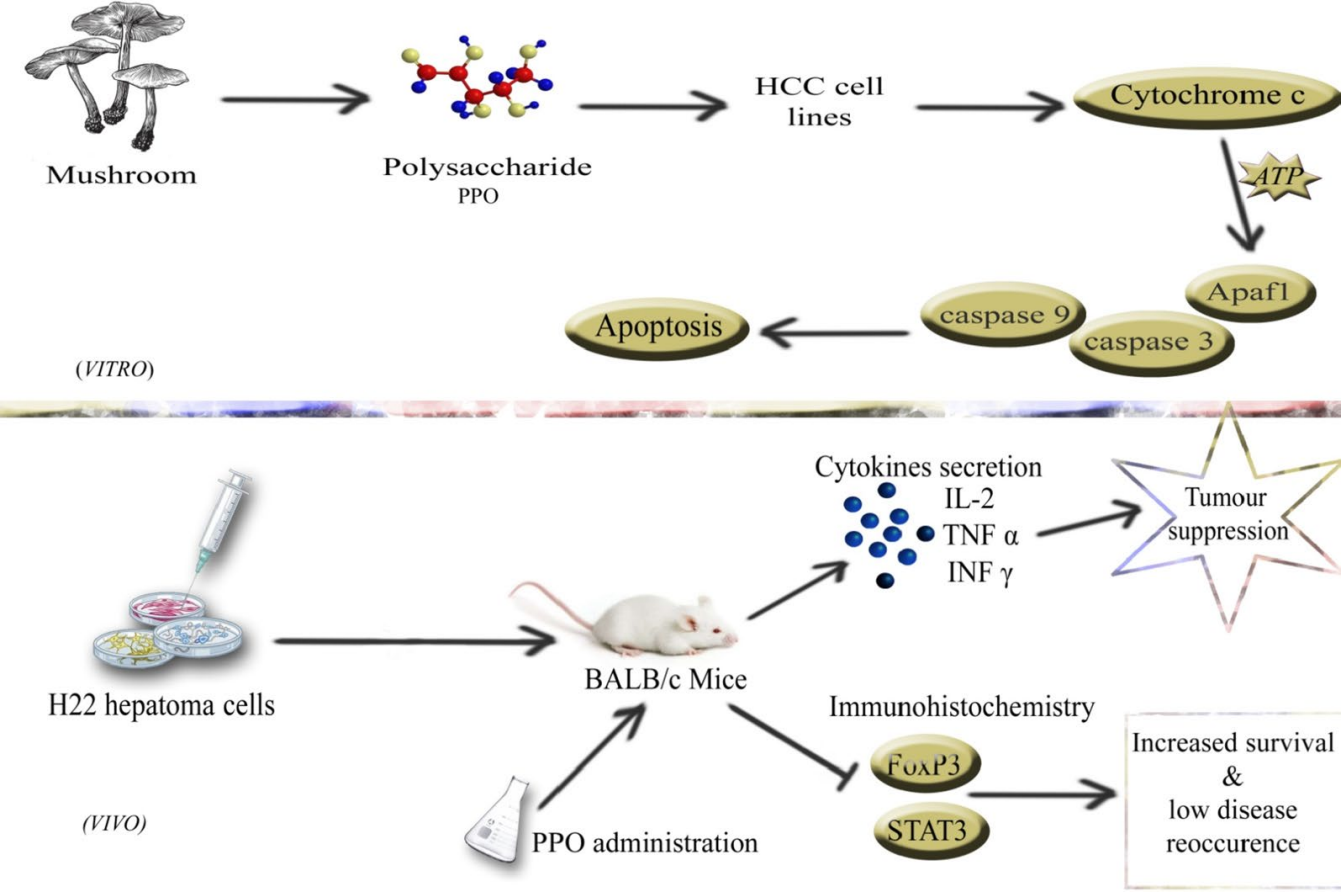
A schematic diagram highlighted the mechanism of polysaccharide extracted from *Pleurotus ostreatus* inducing apoptosis via up-regulation of pro-apoptotic genes and downregulation of anti-apoptotic genes in vitro and explaining how this polysaccharide upon administration enhances the immune response in cancer acquired mice and promotes the secretion of cytokines and regulation of Foxp3 and Stat3 increase survival time and decrease the chances of disease re-occurrence

Genotypic characteristics of hepatocellular carcinoma were observed that shows in Fig. 6 the down-regulation of anti-apoptotic genes and the up-regulation of pro-apoptotic genes. Upon administration of polysaccharide down-regulation of BCL-2 causes the release of Cytochrome-C (Breckenridge and Xue 2004). In Fig. 7 the schematic diagram shows the proposed pathway in which Cytochrome-C protein was assumed to be the initiator of natural cell death as it makes apoptosomes by binding Apaf-1 in the presence of ATP/dATP (Garrido et al. 2006) and following this complex formation, the cascade of caspases activity starts in which caspase-9 is activated (Acehan et al. 2002) and then leads to the expression of Caspase-3 that eventually mediates the biochemical and morphological features of apoptosis. Immuno-fluorescence of Cytochrome C shows the difference between control and treated cells. Pronounced expression of Cytochrome C shows the initiation of the cell death process via apoptosis. Overall polysaccharide from *Pleurotus ostreatus* showed pronounced anti-cancer activity in-vivo and in-vitro experiments. As in-vivo experiments we established a fact that this polysaccharide can act as immunological modulator, similarly in-vitro experiments shows that polysaccharide caused apoptosis initiated by the release of proteins responsible for instigating the cascade of pro-apoptotic proteins. Hence these results shows that this polysaccharide can be used as a potent anti-tumor agent that is why further studies on its chemical characterization is required and our research team is working on specific monomer responsible for anti-cancer activity of polysaccharide.

In conclusion, our investigation reflects that the polysaccharide extracted from *Pleurotus ostreatus* showed anti-tumor activity in-vivo and in-vitro. The polysaccharide in-vivo against H22 malignant ascites tumor cells shows an evident increase in cytokines and decrease in pro-survival proteins in tumor cells that eventually leads to the increasing in the survival period in mice. Coupled with in-vivo results the polysaccharide shows down-regulation of anti-apoptotic proteins and up-regulation of pro-apoptotic genes in cancer cell lines. Therefore, our finding proposed that polysaccharide from *Pleurotus ostreatus* has the potential to be an ideal contender for a more effective strategy against hepatocellular carcinoma.

## Data Availability

The datasets generated during and/or analyzed during the current study are available from the corresponding author on reasonable request.
