# Correction: Anti-tumor effect of polysaccharide from *Pleurotus ostreatus* on H22 mouse Hepatoma ascites in-vivo and hepatocellular carcinoma in-vitro model

**DOI:** 10.1186/s13568-023-01644-6

**Published:** 2023-12-19

**Authors:** Kavish Hasnain Khinsar, Sattar Abdul, Hussain Akbar, Riaz Uddin, Lei Liu, Jing Cao, Majid Abbasi, Ata ur Rehman, Nabeel Farooqui, Xin Yi, Huang Min, Liang Wang, Zhong Mintao

**Affiliations:** 1 Department of Microbiology, College of Basic Medical Sciences, Dalian Medical University, Dalian, 116044 China; 2 Department of Biochemistry, College of Basic Medical Sciences, Dalian Medical University, Dalian, Liaoning, 116044 China; 3 Department of Biotechnology, College of Basic Medical Sciences, Dalian Medical University, Dalian, 116044 China; 4 Department of Rheumatology and Inflammation Research, Institute of Medicine, Sahlgrenska Academy, University of Gothenburg, 43146 Gothenburg, Sweden; 5 Department of Biochemistry, Ghulam Muhammad Mahar Medical College, SMBB Medical University Larkana, Larkana, Pakistan

**Correction: AMB Express (2021) 11:160** **https://doi.org/10.1186/s13568-021-01314-5**

Following publication of the original article (Khinsar et al. 2021), the authors regret for the errors occurred in the figures and funding section. The images of Figs. 3, 4 and 5 have been corrected with this correction and updated Funding information.

**Figure 3A: FoxP3 (c) is similar to FoxP3 (b):**

The following figure shows the IHC images of FoxP3 gene, the (c) part picture is replaced with the correct original one, but it does not make any difference to the statistical analysis, or any other picture associated with the results.

**Fig. 3:**
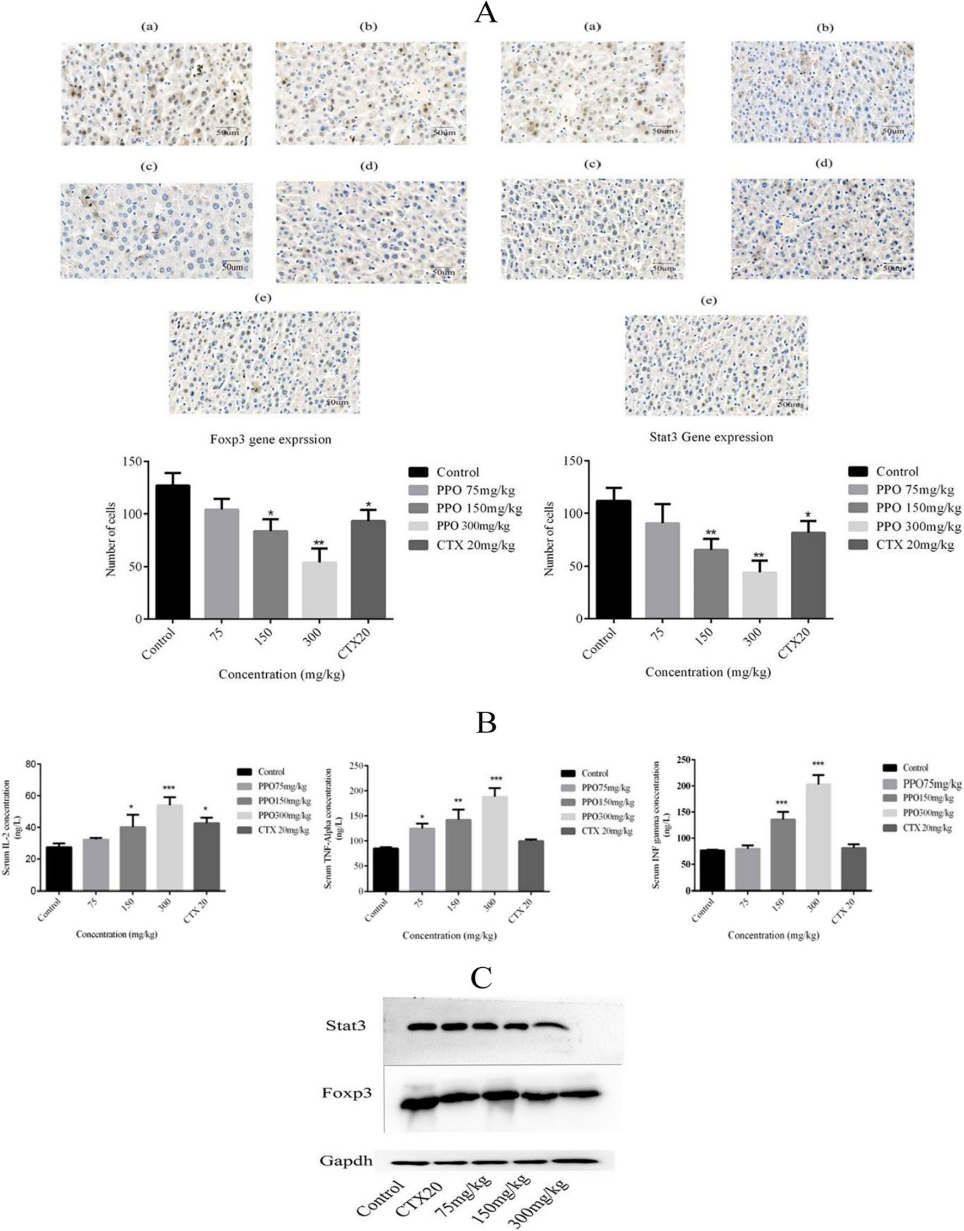
Immunomodulatory effects of polysaccharide on H22 malignant ascites mice model **A** Immunohistochemistry was performed of liver sample tissues to observed Foxp3 and Stat3 expression in mice model and observed a significant decrease in gene expression in a dose-dependent manner. Images were captured at the 40 × lens. **B** Cytokines level was checked by performing Elisa such as IL-2, TNFα, and INFγ was observed to have a significant increase in serum in a dose-dependent manner. **C** Western blot analysis was performed to observe protein expression of Foxp3 and Stat3 and results show a significant dose-dependent decrease in expression. *p < 0.05, **p < 0.01, ***p ≤ 0.001. *PPO (Polysaccharide from *Pleurotus ostreatus*), *CTX (Cyclophosphamide)

**Figure 4B: HepG2 400 µg/ml is similar HepG2 300 µg/ml:**

The following figure shows the colony formation assay for HepG2 cells, the 400 µg/ml picture is replaced with the correct original one, but it does not make any difference to the statistical analysis, or any other picture associated with the results.

**Fig. 4:**
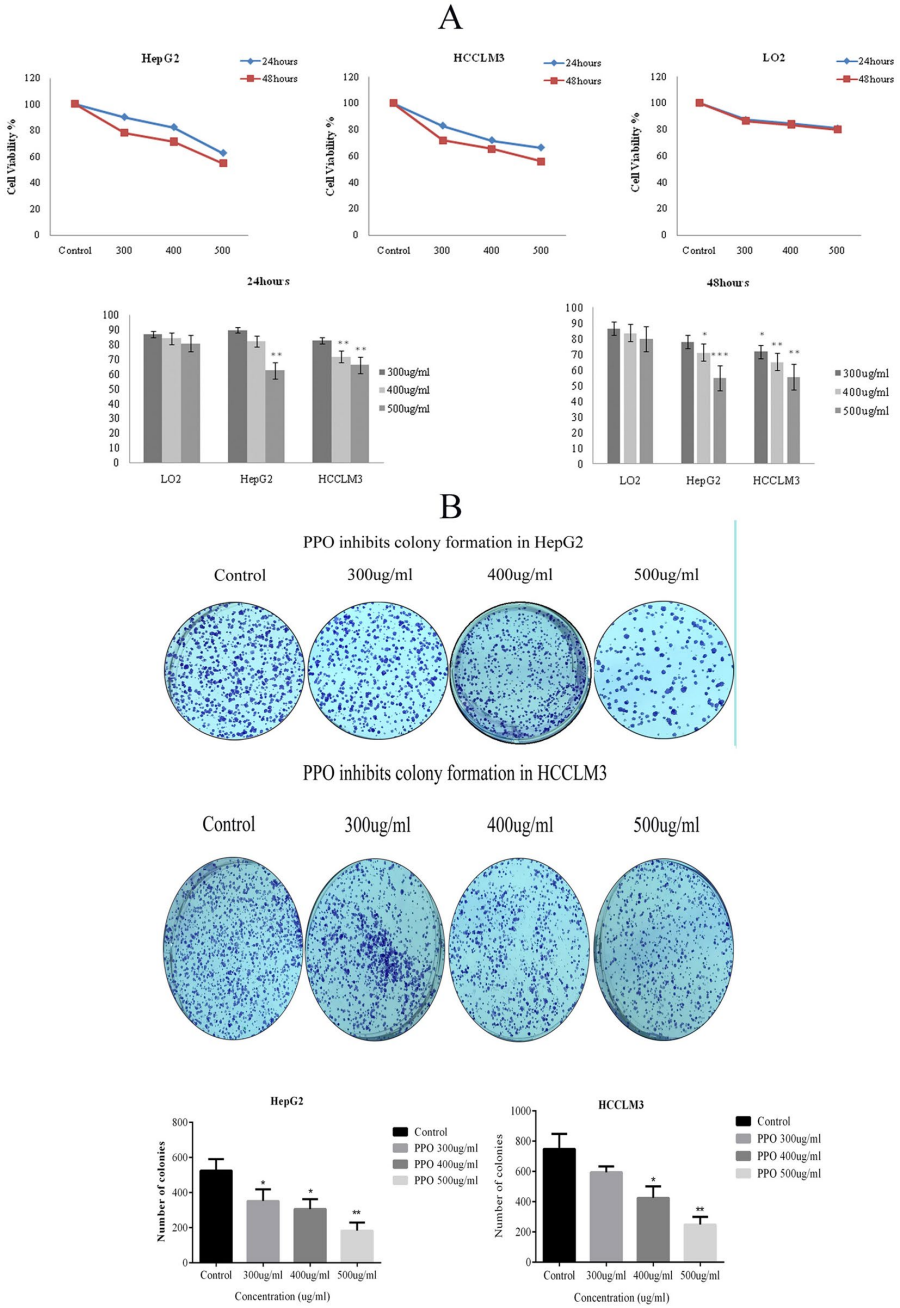
Polysaccharide effects were observed in-vitro using HCC cell lines **A** Cytotoxicity cell viability assay shows the effects of polysaccharide on LO-2, HepG2, and HCCLM3 cell lines. In comparison to normal cell lines, cancer cell lines showed a decrease in cell viability at 24, 48 h with three different concentrations 300, 400, and 500 µg/ml. **B** Polysaccharide inhibits colony formation abilities of cancer cells after treating for 48 h and showed a significant decrease in colony formation abilities of HepG2 and HCCLM3 cell lines in a dose-dependent manner. *p < 0.05, **p < 0.01, ***p ≤ 0.001. *PPO (Polysaccharide from *Pleurotus ostreatus*), *CTX (Cyclophosphamide)

**Figure 5A: HepG2 500 µg/ml 0 h is similar to HepG2 400 µg/ml:**

The following figure shows the wound healing assay for HepG2 cells, the 500 µg/ml, 0-h picture is replaced with the correct original one, but it does not make any difference to the statistical analysis, or any other picture associated with the results. 

**Figure 5A: HCCLM3 400 µg/ml 24 h is similar to 400 µg/ml 48 h:**

The following figure shows the wound healing assay for HCCLM3 cells, the 400 µg/ml, 24-h picture is replaced with the correct original one, but it does not make any difference to the statistical analysis, or any other picture associated with the results. 

**Figure 5C: HCCLM3 500 µg/ml is similar to 400 µg/ml:**

The following figure shows the invasion assay for HCCLM3 cells, the 500 µg/ml, picture is replaced with the correct original one, but it does not make any difference to the statistical analysis, or any other picture associated with the results.

**Fig. 5:**
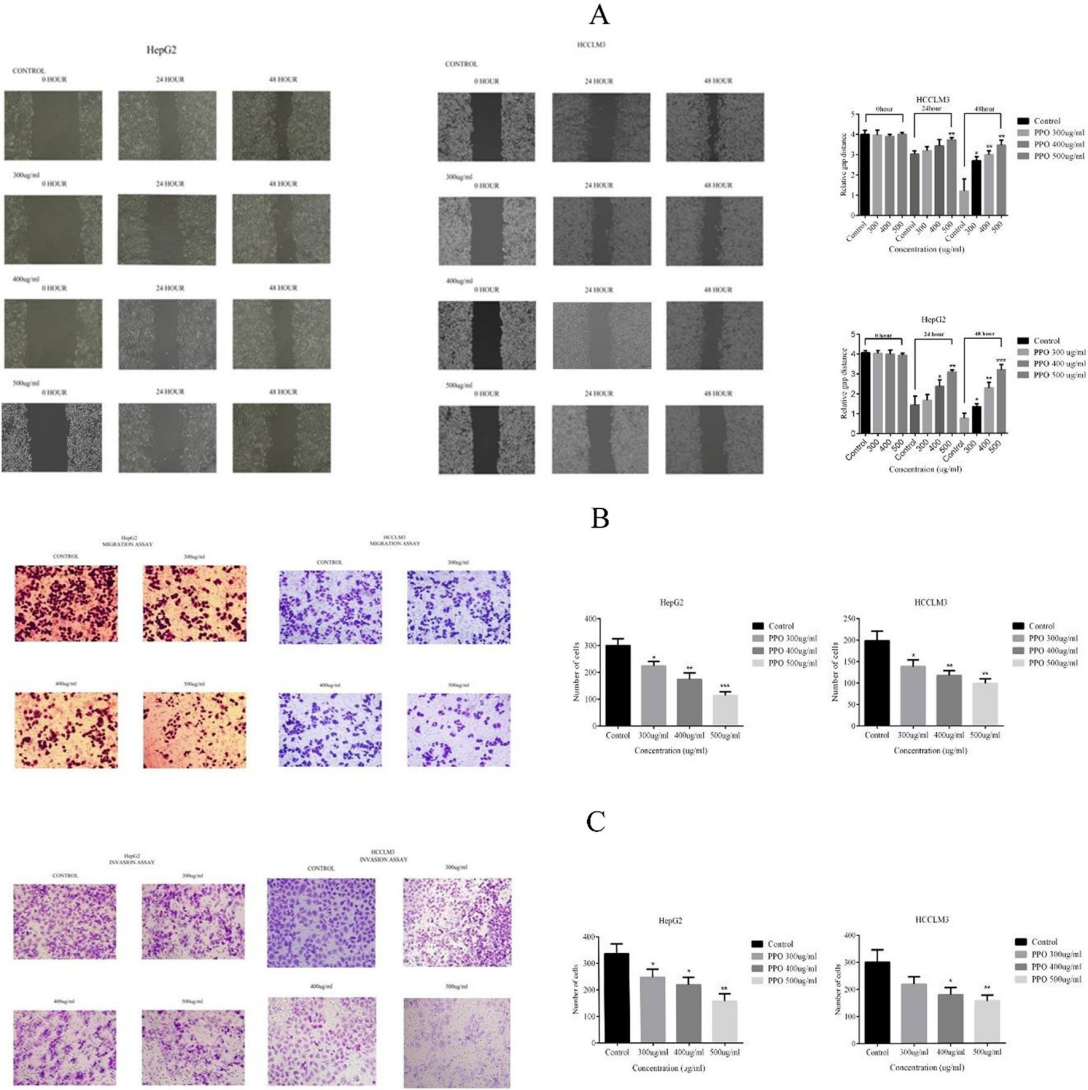
Polysaccharide affects anti-metastatic abilities of cancer cells **A** Wound closure assay was performed and observe the significant difference between Control and polysaccharide treated cells after comparing relative gap distance in HepG2 and HCCLM3. **B** To observe the anti-migratory abilities of polysaccharide in HepG2 and HCCLM3, transwell chambers assay was performed and the difference was observed in decrease number of migrated cells in a dose-dependent manner **C** Transwell chamber was used for invasion assay and the results suggest that there is a significant difference as compared to control group and observed a decrease in the invaded number of cells in a dose-dependent manner. *p < 0.05, **p < 0.01, ***p ≤ 0.001. *PPO (Polysaccharide from *Pleurotus ostreatus*), *CTX (Cyclophosphamide)

Under Funding paragraph, the authors want to remove the grant numbers (81301995; 81472836) and replaced it with Chinese Government Scholarship (CSC no. 2015GXZ482). The correct Funding should appear as:

This study was supported by Grants from The National Natural Science Foundation of China (31600614, 82072953), Top Young talents of Liaoning Provincial Government (XLYC1907009). Chinese Government Scholarship (CSC no. 2015GXZ482).
